# Trends in Patient Characteristics and Cardiothoracic Surgeries over 14 Years (2010–2023): A Single Center Experience

**DOI:** 10.3390/jcm13216467

**Published:** 2024-10-28

**Authors:** Orr Sela, Sergei Gelman, Amit Gordon, Ariel Farkash, Dmitri Pevni, Mohammad Kakoush, Jonathan Kfir, Yanai Ben-Gal

**Affiliations:** Department of Cardiothoracic Surgery, Tel Aviv Sourasky Medical Center and Faculty of Medicine, Tel Aviv University, Tel-Aviv 6423906, Israel; orrse@tlvmc.gov.il (O.S.); sergeig@tlvmc.gov.il (S.G.); amitgor@tlvmc.gov.il (A.G.); arielfa@tlvmc.gov.il (A.F.); dimitrip@tlvmc.gov.il (D.P.); mohammadk@tlvmc.gov.il (M.K.); jonakfir@berkeley.edu (J.K.)

**Keywords:** coronary artery disease, CABG, coronary artery bypass surgery, valvular disease, aortic valve, AVR, MVR, PTCA, transcatheter valve replacement

## Abstract

**Background:** as transcatheter technologies have advanced, the patient population that is referred to open heart surgeries has shifted. This study’s objective was to evaluate recent trends in the characteristics of patients undergo surgical valvular interventions and coronary revascularizations (CABG) in our center over a period of 14 years. **Methods:** this is a retrospective analysis of ecological trends in the age, sex, and risk profile (Charlson comorbidity index—CCI) of patients who, from January 2010 to December 2023, underwent CABG, aortic valve replacement (AVR), or mitral valve repair or replacement (with or without tricuspid valve intervention). The data were extracted from electronic clinical files using MD-Clone software. **Results:** for the CABG procedures, the respective data for 2010 and 2023 were: mean ages 68.0 and 64.6 years; 79.7% and 83.1% males; and mean CCI scores 3.16 and 2.51. The *p*-values for the cumulative differences over the study period were 0.001, 0.005, and 0.013, respectively. The respective data for isolated AVR were mean ages of 69.2 and 62.9 years; 64.1% and 59.1% males; mean CCI 3.64 and 2.32; *p*-values: <0.001, 0.229, and 0.019. The respective data for mitral valve procedures were mean ages of 63.6 and 59.8 years, 71.4% and 65.5% males; mean CCI 2.90 and 1.79; *p*-values: 0.84, 0.422, and 0.318. **Conclusions:** over a 14-year period, changes were evident in the age, sex distribution, and CCI for operations performed in our center. These changes most likely resulted from accumulated data regarding the advantages and detriments of treatment strategies, mostly of CABG vs. percutaneous coronary intervention; major advancements in transcatheter technologies, mostly in transcatheter AVR; and clinical guidelines facilitating a more collaborative decision-making, open-minded, and personalized approach.

## 1. Introduction

Cardiothoracic surgeries, and particularly their main stem procedures (coronary artery bypass graft (CABG) surgery, aortic valve replacement (AVR) and mitral valve repair or replacement (MVR)), are critical interventions for managing prominent cardiovascular diseases. Over the past two decades, the landscape of these surgical procedures has evolved significantly, consequent to innovations in the fields of minimally invasive techniques, transcatheter interventions, medical technologies, medical treatments, and postoperative care. In parallel, transcatheter advancements and, in particular, percutaneous coronary interventions (PCIs) that deploy drug eluting stents (DESs), transcatheter AVR, and edge-to-edge transcatheter MVR, provide a wider array of options for patients with cardiovascular diseases. In addition, more collaborative decision-making processes have prompted both cardiologists and cardiothoracic surgeons to tailor treatment strategies to their patients [1,2,3,4]. This multidisciplinary approach aims for up-to-date, educated, and individualized treatment-strategy decisions which consider the patient’s medical background, the complexity of the disease, its anatomic features, and other patient-specific factors. It remains to be seen if all these technological advances, together with the multidisciplinary referral patterns (i.e., “heart teams”), have resulted in improved long-term outcomes and an enhanced overall quality of life for these patients. The aim of this retrospective ecologic analysis was to evaluate or “snapshot” the basic epidemiologic shift in the patient populations that were referred to the main surgical pathways of CABG, AVR, and MVR operations.

## 2. Materials and Methods

### 2.1. Patients and Methods

Tel Aviv Sourasky Medical Center is a major tertiary referral center in Tel Aviv, Israel, with a heart center dedicated to the treatment of cardiovascular diseases. Annual data from the last two decades show more than 20,000 cardiology clinic visits, 17,000 transthoracic and transesophageal echocardiograms, 5000 diagnostic and interventional cardiac catheterization procedures, 300 transcatheter valve procedures, and 600 adult open heart surgery procedures.

This ecological study comprised all the patients who underwent isolated CABG, AVR, or MV repair or replacement (with or without tricuspid valve interventions) in Tel-Aviv Medical Center during 2010–2023. A separate analysis was conducted of patients who underwent a multivalve procedure, such as an AVR together with either an MVR or a tricuspid valve procedure. Patients who underwent other procedures such as operations involving the aorta or other structural or hybrid procedures were excluded from the analysis, as were those who underwent a CABG procedure combined with valve procedures. This study was approved by our institutional review board (approval number: 0146-14-TLV) and the data protection officer in our institution. Informed consent was waived due to the retrospective study design.

Data regarding the sex, age, and Charlson comorbidity index (CCI) were collected using MD-Clone software, which extracts data from electronic admission files and hospital clinics, including cardiothoracic surgery and cardiology clinics. MD-Clone is a healthcare data-exploration platform that automatically extracts multiple demographic, clinical, and laboratory variables from electronic health records. It can also calculate variables such as early mortality (within 30 days of the procedure) and other early outcomes [5].

CCI is a prevalent method of classifying the comorbidity of cardiac patients. It provides a simple, readily applicable, and valid method to estimate the risk of death from comorbidities, and is frequently used in longitudinal studies [6]. Due to the wide use of CCI in the field as a common method to assess the added risk of comorbidities, it has been validated [7,8]. The study inclusion criteria were: (i) age above 18 years; (ii) having undergone isolated CABG or isolated AVR or isolated mitral valve repair or replacement with or without tricuspid valve intervention, or having undergone a multiple valve procedure.

In principle, during the study period, the preference to refer patients for surgical procedures was in accordance with the general guidelines of the American College of Cardiology and the European Society of Cardiology [1,2,3,4]. Patients were admitted either directly from in-house cardiology or internal medical wards, or as an outside referral from out-of-hospital cardiology clinics or hospitals from the periphery.

### 2.2. Statistical Analysis

Age and CCI scores were reported as means and standard deviations (SDs), while patient sex was reported as number and percentage. These parameters were summarized for each of the study years. Spearman’s rank correlation coefficient was used to evaluate the strengths and the directions of the monotonic trends during the study period. Spearman’s rank correlation analysis was chosen as this approach is less restrictive than measuring the linear relation by linear regression. The use of Spearman’s rank correlation coefficient to detect trends was described previously [9]. In the current study, the year of the procedure was considered as an interval variable, while the mean age, the proportion of males, and the mean CCI scores were considered as ratio variables. A two-tailed *p*-value of 0.05 was considered significant. SPSS software was used for the statistical analysis (IBM SPSS Statistics for Windows, ver. 29, IBM corp., Armonk, NY, USA, 2023).

## 3. Results

During the designated period, a total of 3287 patients underwent isolated CABG procedures, 700 underwent isolated AVRs, 497 underwent isolated MVR procedures (with or without tricuspid valve repair or replacement), and 224 underwent multivalve procedures (involving the aortic and mitral valve together).

### 3.1. CABG

**Figure 1:**
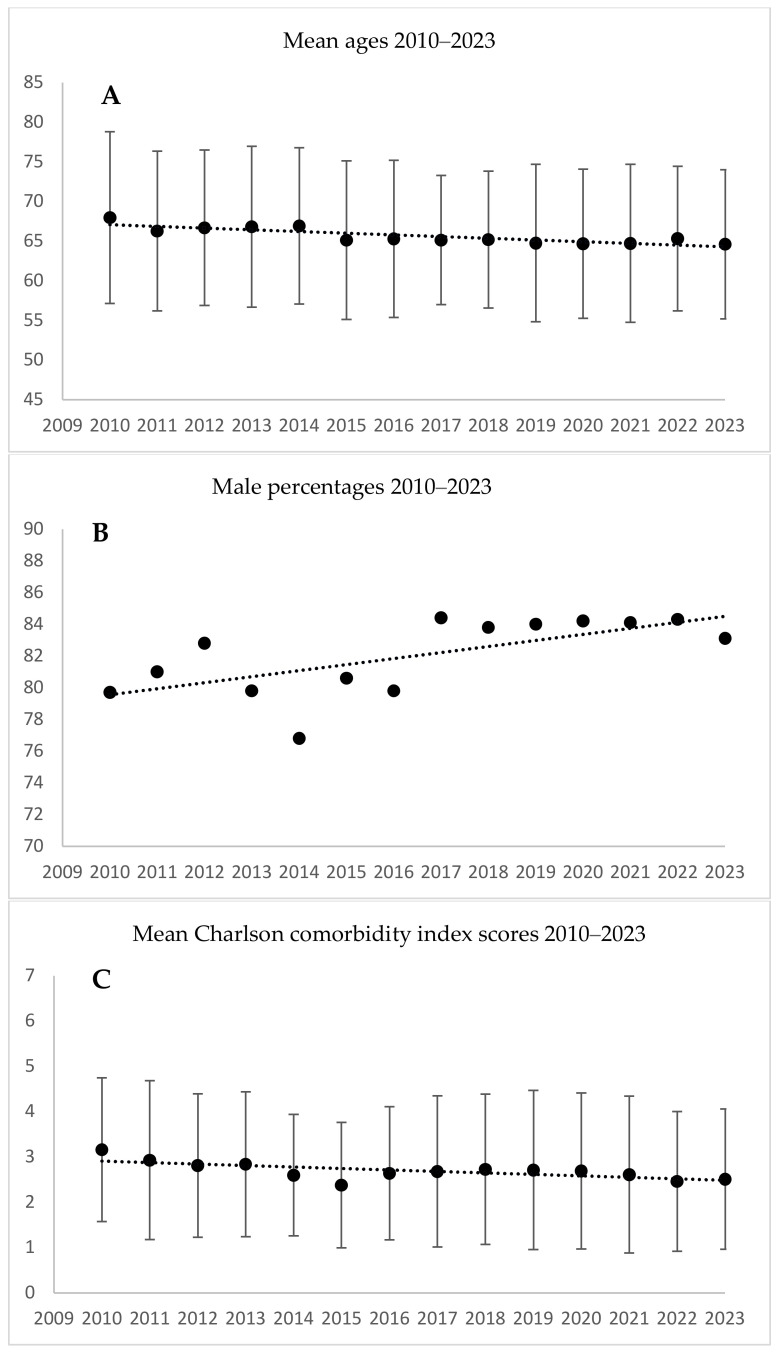
Coronary revascularization (CABG) procedures: patient age, male proportion, and Charlson comorbidity index scores during 2010–2023. (**A**) CABG ages: correlation coefficient = −0.85, *p* = 0.001; (**B**) CABG male proportion: correlation coefficient = 0.666, *p* = 0.005; (**C**) CABG Charlson comorbidity index: *p* = 0.013. The linear trend line is only for illustration. The *p*-values represent the significance of the monotonic trend over 14 years as measured by Spearman’s rank correlation analysis.

In 2010, the mean age of the patients who underwent CABG was 68.0 years, 79.7% were males, and the mean CCI score was 3.16. In 2023, the mean age was 64.6 years, 83.1% were males, and the mean CCI score was 2.51 (Table 1, Figure 1). The cumulative differences were significant for age (*p* = 0.001), sex (*p* = 0.005), and the CCI score (*p* = 0.013). For each year in the study period, the number of patients, the mean age, the male proportion, and the mean CCI scores are summarized in Table 1. The trends in the three parameters are depicted in Figure 1.

### 3.2. AVR

**Figure 2:**
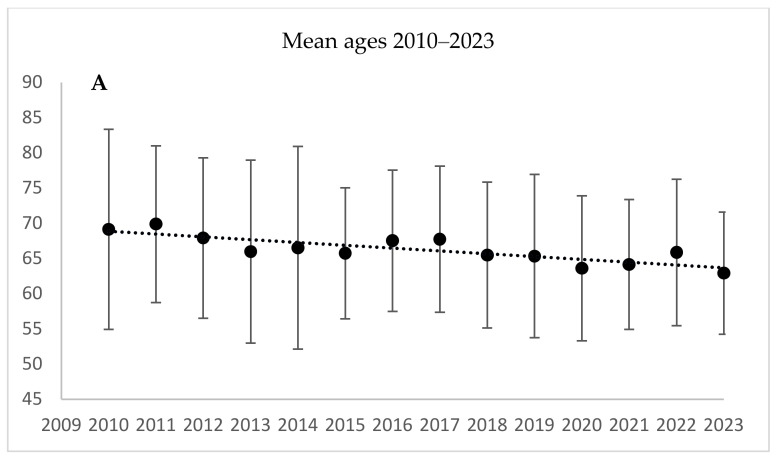

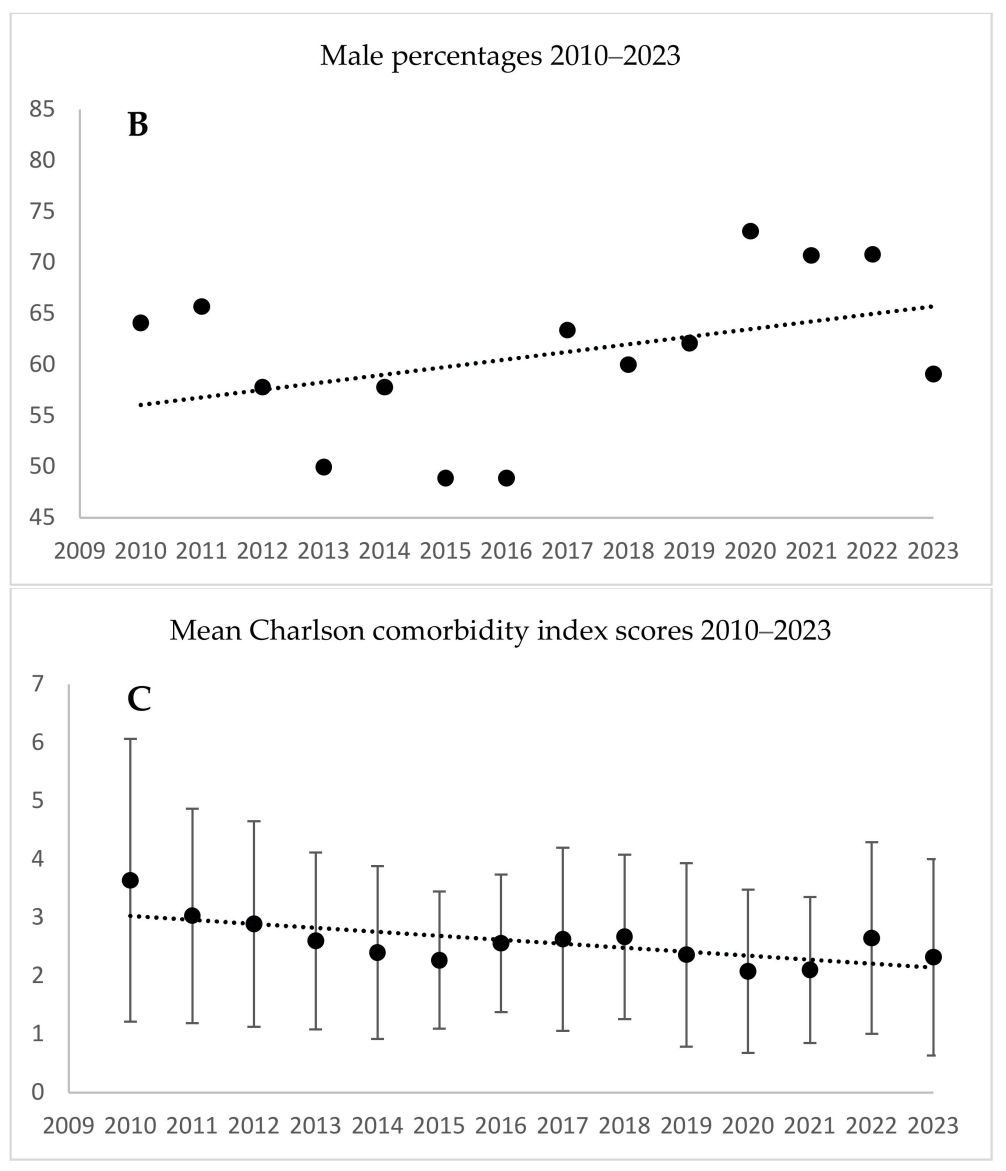
Aortic valve replacement procedures (AVRs): patient age, male proportion, and Charlson comorbidity index scores during 2010–2023. (**A**) AVR ages: correlation coefficient = −0.832, *p* < 0.001; (**B**) AVR male proportion: correlation coefficient = 0.393, *p* = 0.229; (**C**) AVR Charlson comorbidity index: *p* = 0.019. The linear trend line is only for illustration. The *p*-values represent the significance of the monotonic trend over 14 years as measured by Spearman’s rank correlation analysis.

In 2010, the mean age of the patients who underwent AVR was 69.2 years, 64.1% were male, and the mean CCI score was 3.64. In 2023, the mean age was 62.9 years, 59.1% were male, and the mean CCI score was 2.32. The cumulative differences were non-significant for the sex distribution (*p* = 0.229), but were significant for age and CCI (*p* < 0.001 and *p* = 0.019, respectively) (Table 2, Figure 2).

### 3.3. MVR

**Figure 3:**
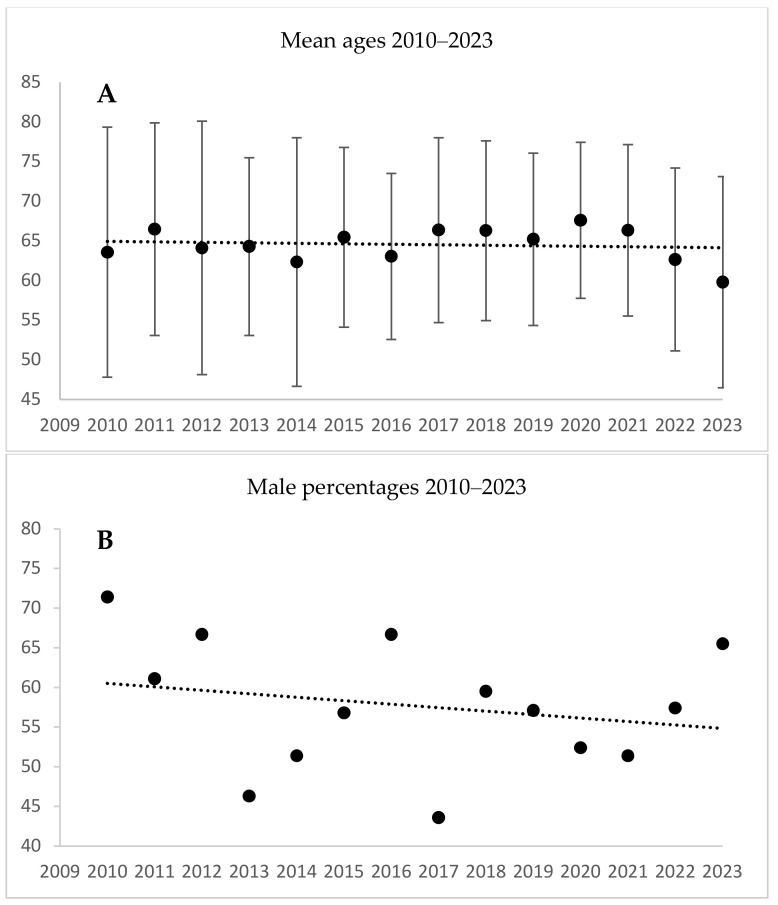

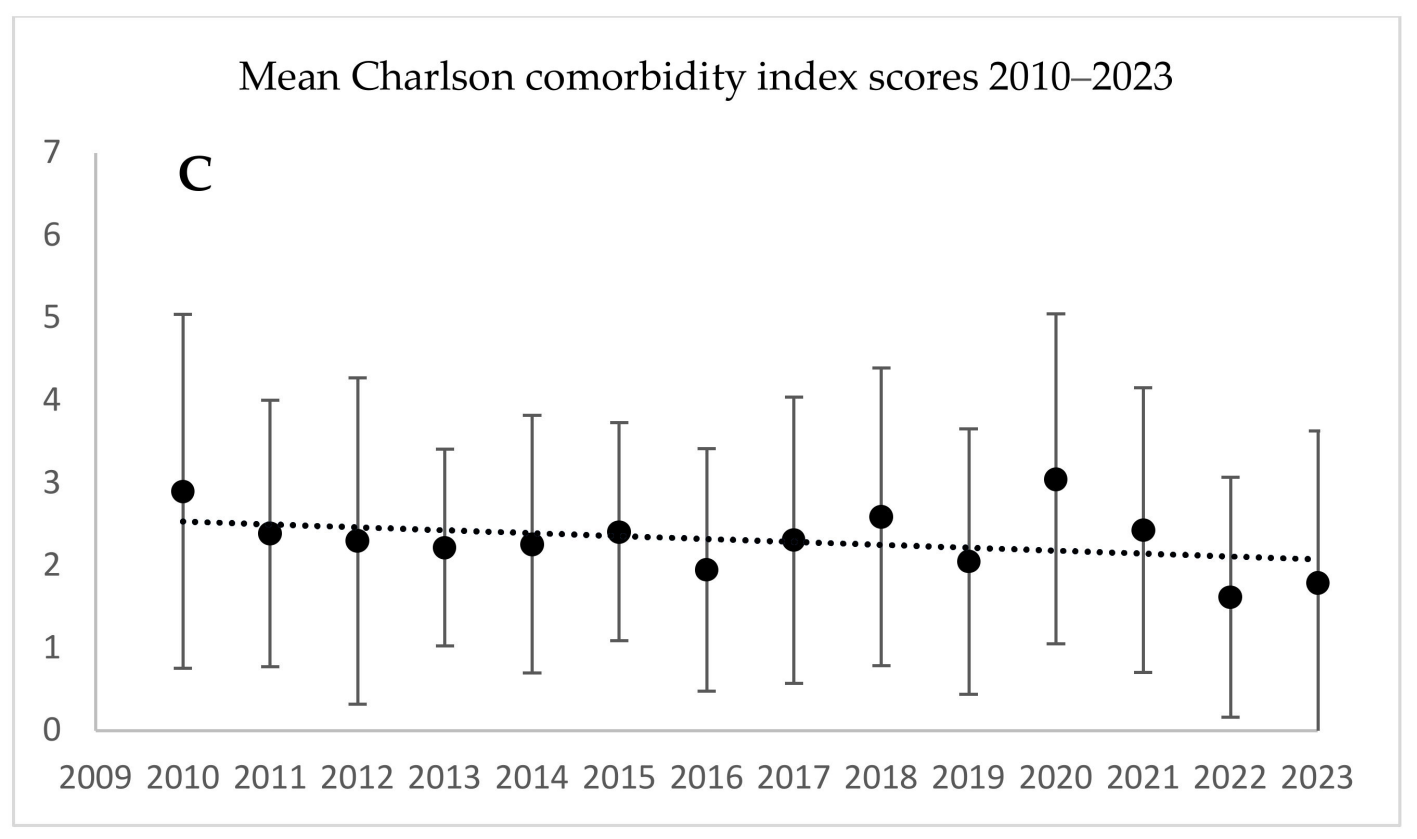
Mitral valve repair or replacement procedures (MVR): patient age, male proportion, and Charlson comorbidity index scores during 2010–2023. (**A**) MVR ages: correlation coefficient = −0.578, *p* = 0.84; (**B**) MVR male proportion: correlation coefficient = −0.224, *p* = 0.422; (**C**) MVR Charlson comorbidity index: *p* = 0.318. The linear trend line is only for illustration. The *p*-values represent the significance of the monotonic trend over 14 years as measured by Spearman’s rank correlation analysis.

For isolated MVR, the respective data for 2010 and 2023 were mean ages of 63.6 and 59.8 years, male proportions of 71.4% and 65.5%, and mean CCI scores of 2.9 and 1.79. The respective *p*-values were 0.840, 0.422, and 0.318 for the cumulative period (Table 3, Figure 3).

### 3.4. Multivalve Procedures

**Figure 4:**
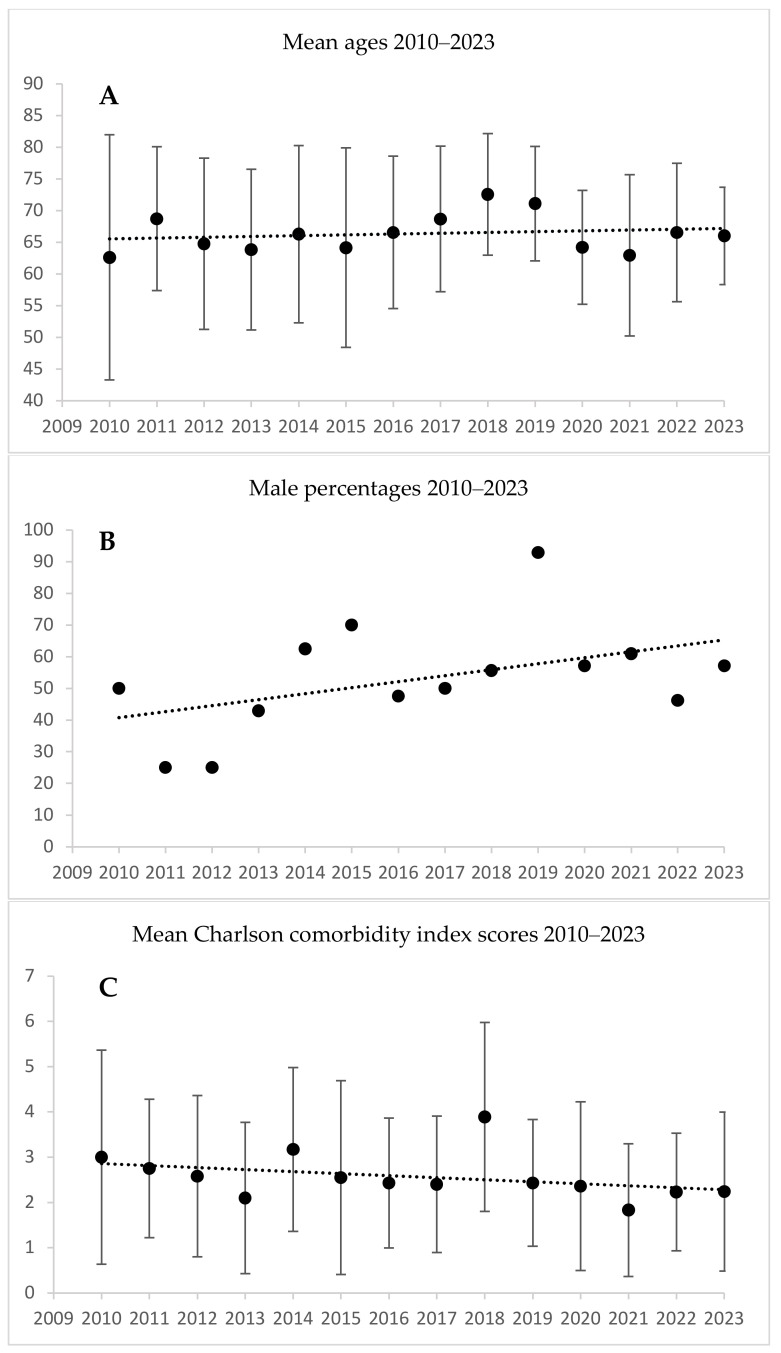
Multivalve procedures: patient age, male proportion, and Charlson comorbidity index scores during 2010–2023. (**A**) Multivalve ages: correlation coefficient = 0.178, *p* = 0.553; (**B**) multivalve male proportion: correlation coefficient = 0.46, *p* = 0.127; (**C**) multivalve Charlson comorbidity index: *p* = 0.028. The linear trend line is only for illustration. The *p*-values represent the significance of the monotonic trend over 14 years as measured by Spearman’s rank correlation analysis.

For multivalve (aortic and mitral valve procedures, with or without tricuspid repair) procedures, the mean CCI score was higher in 2010 than in 2023: 3.00 vs. 2.24, *p* = 0.028. The mean age and proportion of males did not differ over the cumulative period (Table 4, Figure 4).

## 4. Discussion

We report that the demographic profile of patients who underwent the most prominent surgical cardiac procedures in our center during the last 14 years evolved markedly. This shift can be attributed to several factors, such as the aging population and the increased prevalence of cardiovascular risk factors. However, we assume it is mostly consequent to the publication of studies that evaluated differences between interventional and surgical strategies, together with the ongoing advancements in transcatheter coronary and valvular interventions. We suggest a number of reasons for the observed trend of a younger and less morbid population that underwent CABG and surgical AVR procedures. Compared to PCIs, CABG procedures tended to be performed at earlier stages due to the demonstrated long-term benefit of surgical outcomes. In parallel, surgical AVRs tended to be performed in younger and healthier patients due to improvements in transcatheter aortic valve interventions, which were targeted during the study period towards older and sicker populations.

As expected, we did not find significant demographic changes in patients who underwent surgical mitral valve procedures during the study period. This is apparently because the transcatheter advancements and the literature evaluating them were not as distinct as in the coronary and aortic valve territories.

### 4.1. Demographic Changes in the Patients Who Underwent CABG Surgery

With the introduction of DESs in the first decade of the 2000s, the demographic profile of patients who underwent CABG shifted towards an older and more comorbid population. An initial decline in the number of CABG procedures was observed, as younger lower-risk patients were more often managed with PCI, while surgery was performed mostly in patients with more complex coronary disease who were deemed unsuitable for PCI [10,11,12,13]. The rise in the performance of PCIs has unquestionably affected CABG rates. The SYNTAX trial demonstrated that, for certain patient subsets, PCI with DES offered comparable outcomes to CABG, thus leading to a shift in treatment strategies [14]. Despite this shift, and in contrast with the trend described above, CABG surgery continued to demonstrate superior long-term survival benefits compared to PCI. Indeed, later publications demonstrated the long-term efficacy of CABG compared to multivessel PCI with DES. As a result, the trend has shifted back to early surgical intervention, including for younger and healthier patients [15,16,17,18,19]. Regarding gender, the trend has shifted towards a higher male percentage in recent years (CABG male proportion: *p* = 0.005). We postulate that this is related to the shift in the referral pattern for surgery in younger and “healthier” patients (with lower Charlson score indices). The same is applicable for the female gender, which is recognized as a risk factor for both CABG and PCI [20,21]. Consequently, male patients (at lower risk for either intervention, PCI or CABG) began to be referred in higher numbers for the “more comprehensive” revascularization strategy. On the other hand, referral for PCI was reserved for patients with higher risk features, who were mostly older, with higher Charlson scores, and predominantly of the female gender. Therefore, even in the current era of DES, CABG surgery remains a cornerstone for treating coronary artery disease, particularly for patients with diabetes mellitus and complex multivessel lesions.

In addition to the conventional CABG procedure, which employs single internal thoracic artery (ITA) grafting and saphenous vein grafts, bilateral ITA grafting offers unique advantages, including better long-term survival than single ITA with venous graft combinations. Moreover, bilateral ITA grafting, compared to single ITA grafting, has shown a reduced risk of myocardial infarction and other major adverse coronary events. This emphasizes the role of CABG surgeries in optimizing long-term outcomes [22,23,24]. We presume that the routine implementation of bilateral ITA revascularization in our center enhanced the advantages of surgical revascularization beyond those that were depicted in recent studies, with the latter causing a shift towards younger and “healthier” patients, a lower proportion of whom are females (a known risk factor for CABG), and lower mean CCI scores. We assume that our cardiologists acknowledged our BITA strategy by increasing their tendency to refer even younger and “healthier” patients to surgical revascularization.

### 4.2. Demographic Changes in Surgical AVR

As the standard treatment for severe aortic valve diseases, surgical AVR offers durable outcomes and improved survival rates. Notably, advancements in surgical techniques, such as minimally invasive strategies, have reduced the procedural discomfort and the need for blood products, and shortened the recovery times. Nonetheless, the introduction and rapid adoption of transcatheter aortic valve implantation (TAVI) has substantially influenced the treatment landscape of aortic stenosis.

Initially, transcatheter strategies expanded the treatment options for populations that were considered as having a high operative risk. This altered the overall patient demographics and clinical outcomes that were associated with aortic valve interventions [25]. Further, studies like PARTNER 2, PARTNER 3, SURTAVI, and the EVOLUTE low risk trial [25,26,27,28] highlighted TAVI procedures as a potential alternative to surgical AVR for the short and intermediate term in intermediate-risk and even in properly selected low-risk patients. These changes significantly influenced the referral patterns of patients with severe symptomatic aortic stenosis. Currently, older and higher-risk patients are increasingly managed with TAVI, while only younger and healthier patients are referred to surgical AVR [29,30,31,32]. This trend was evidenced in our center by the statistically significant decline in the mean age and mean CCI scores of the patients who underwent surgical AVR. The medical logic for this trend in surgical AVR was the same as for patients with coronary artery disease [26,29,30,31,32,33]. Again, we are still in the midst of the paradigm shift and the appropriate strategy to intervene in these patients with intermediate- and low-risk severe aortic stenosis is yet to be determined.

### 4.3. Demographic Changes in Surgical MV Repair or Replacement Procedures

The field of transcatheter MVR procedures has also shown significant progression in recent years, particularly with the development and refinement of transcatheter edge-to-edge repairs (TEER). These advancements, together with progress in surgical MVR procedures, have introduced new populations in need of MVR interventions. Many patients once deemed unsuitable for any intervention other than medical therapy are currently referred to minimally invasive surgical repairs or replacements, and mostly to TEER. Again, the selection of the appropriate strategy, whether TEER or surgery, is still evolving. Ongoing and future studies may further elucidate the advantages and limitations of each therapeutic approach.

In contrast to the trends described previously, surgical MVR remains the standard for most patients due to its definite nature and proven success and durability. Moreover, data are limited regarding the efficacy and durability of transcatheter interventions. TEER and balloon mitral valve valvuloplasty are still unsuitable procedures for many patients and pathologies, either due to comorbidities necessitating surgical intervention (severe CAD for example) or due to various morphologic features. Examples of the latter are retracted leaflets, calcified mitral apparatuses, and the mixed pathology of stenosis with regurgitation. This trend is even more pronounced in pathologies of the tricuspid valve, for which the data are particularly scarce. Consistent with these findings, we report unchanged demographic and risk characteristics for patients who were referred to surgical mitral (with or without tricuspid) interventions in the last decade, as well as for patients in need of multiple valvular interventions. The latter also include the mitral or tricuspid valves, in addition to the aortic valve.

### 4.4. Limitations

As a single-center observational retrospective analysis, this study bears some inherent limitations. First, due to the prolonged follow-up, a calendar bias cannot be ruled out. Personnel changes in both the surgical team and the cardiology department may have influenced referral preferences alongside global trends and advancements. Due to the long follow-up, some data were unattainable or were retrieved mostly from concise preoperative data recorded at admission or surgical or catheterization reports regarding preoperative coronary lesions and valvular morphology. Examples of the latter are bicuspid vs. tricuspid aortic stenosis and mitral annular calcifications leading to non-surgical procedures. Moreover, surgical AVR procedures were not classified according to the main pathophysiological entity of stenosis or regurgitation. TAVR, the competing strategy for surgical AVR, is mostly inapplicable for pure aortic insufficiency and is indicated for only a small minority of AVR cases. Consequently, important data regarding the logic or the reasoning behind the referral preferences for these rare cases were unavailable. This may have impinged on our interpretation of the data. Additionally, no strict guidelines dictated the procedural preference and not all the patients were evaluated by a full-scale heart team, either due to urgency considerations or for no apparent reason. Thus, treatment allocation bias cannot be ruled out. Finally, our study focused on surgical patients only, with no assessment or evaluation of patients who underwent transcatheter interventions. This impedes reaching a clear conclusion regarding non-surgical referral patterns and interdisciplinary trends in the past decade.

## 5. Conclusions

In our 14-year study, CABG surgery demonstrated a viable alternative compared to PCI for patients with multivessel coronary disease. This may be due to its proven superiority in terms of survival, reinterventions, and late myocardial infarctions, as manifested in the vast literature of recent years. While both surgical AVR and TAVI provide practical alternatives for patients with severe aortic stenosis, surgical AVR remains the first option for younger and healthier patients with longer life expectancy, while TAVR has gained its place for the older and sicker population. This explains the shift towards younger and healthier patients being referred for surgical AVR. The referral patterns for surgical mitral MVR interventions have remained unchanged. This may be due to the insufficient advancement of transcatheter technologies in this field and the scarce data regarding their long-term benefits.

A thoughtful understanding of the above trends in clinical practice can contribute to establishing more collaborative decision-making, as recommended by current clinical guidelines, and facilitate a more personalized approach to treatment strategies. Educated patient referral, which balances the precise risks and benefits of the available interventions, can benefit clinicians and health care managers, regulators, industry leaders, and program directors, and help prepare for future trends in the cardiovascular domain. As advancements in medical technology and surgical techniques continue to evolve, ongoing research and clinical trials will be essential for the further refinement of referral practices in cardiac surgery and interventional cardiology, ultimately improving patients’ survival and quality of life.

## Figures and Tables

**Table 1:** Coronary revascularizations (CABG) procedures during 2010–2023.

Year	All CABG	Male (%)	Age, Mean (SD)	CCI, Mean (SD)
2010	177	141 (79.7%)	68.0 (10.8)	3.16 (1.585)
2011	158	128 (81.0%)	66.3 (10.1)	2.93 (1.753)
2012	192	159 (82.8%)	66.7 (9.8)	2.81 (1.584)
2013	178	142 (79.8%)	66.8 (10.2)	2.84 (1.599)
2014	181	139 (76.8%)	66.9 (9.9)	2.60 (1.341)
2015	222	179 (80.6%)	65.1 (10.0)	2.38 (1.385)
2016	253	202 (79.8%)	65.3 (9.9)	2.64 (1.470)
2017	270	228 (84.4%)	65.1 (8.2)	2.68 (1.666)
2018	278	233 (83.8%)	65.2 (8.6)	2.73 (1.659)
2019	256	215 (84.0%)	64.8 (9.9)	2.71 (1.756)
2020	265	223 (84.2%)	64.7 (9.4)	2.69 (1.720)
2021	290	244 (84.1%)	64.7 (10.0)	2.61 (1.730)
2022	306	258 (84.3%)	65.3 (9.1)	2.46 (1.543)
2023	261	217 (83.1%)	64.6 (9.4)	2.51 (1.546)
Sum	3287			
*p*-value		0.005	0.001	0.013

SD, standard deviation; CCI, Charlson comorbidity index. The *p*-values represent the significance of the monotonic trend over 14 years as measured by Spearman’s rank correlation analysis.

**Table 2:** Aortic valve replacement (AVR) procedures during 2010–2023.

Year	All AVR	Male (%)	Age, Mean (SD)	CCI, Mean (SD)
2010	39	25 (64.1%)	69.2 (14.2)	3.64 (2.422)
2011	35	23 (65.7%)	69.9 (11.1)	3.03 (1.839)
2012	45	26 (57.8%)	67.9 (11.4)	2.89 (1.761)
2013	42	21 (50.0%)	66.0 (13.0)	2.60 (1.515)
2014	45	26 (57.8%)	66.5 (14.4)	2.40 (1.483)
2015	45	22 (48.9%)	65.8 (9.3)	2.27 (1.176)
2016	50	29 (58.0%)	67.5 (10.0)	2.56 (1.181)
2017	71	45 (63.4%)	67.7 (10.4)	2.63 (1.570)
2018	60	36 (60.0%)	65.5 (10.4)	2.67 (1.410)
2019	66	41 (62.1%)	65.4 (11.6)	2.36 (1.575)
2020	52	38 (73.1%)	63.6 (10.3)	2.08 (1.398)
2021	58	41 (70.7%)	64.2 (9.2)	2.10 (1.252)
2022	48	34 (70.8%)	65.9 (10.4)	2.65 (1.644)
2023	44	26 (59.1%)	62.9 (8.7)	2.32 (1.681)
Sum	700			
*p*-value		0.229	<0.001	0.019

SD, standard deviation; CCI, Charlson comorbidity index. The *p*-values represent the significance of the monotonic trend over 14 years as measured by Spearman’s rank correlation analysis.

**Table 3:** Mitral valve repair or replacement (MVR) procedures during 2010–2023.

Year	All MVR	Male (%)	Age, Mean (SD)	CCI, Mean (SD)
2010	21	15 (71.4%)	63.6 (15.8)	2.90 (2.143)
2011	18	11 (61.1%)	66.5 (13.4)	2.39 (1.614)
2012	33	22 (66.7%)	64.1 (16.0)	2.30 (1.976)
2013	41	19 (46.3%)	64.3 (11.2)	2.22 (1.194)
2014	35	18 (51.4%)	62.3 (15.7)	2.26 (1.559)
2015	37	21 (56.8%)	65.5 (11.3)	2.41 (1.322)
2016	39	26 (66.7%)	63.0 (10.5)	1.95 (1.468)
2017	39	17 (43.6%)	66.4 (11.7)	2.31 (1.734)
2018	37	22 (59.5%)	66.3 (11.3)	2.59 (1.802)
2019	42	24 (57.1%)	65.2 (10.9)	2.05 (1.607)
2020	42	22 (52.4%)	67.6 (9.8)	3.05 (1.999)
2021	37	19 (51.4%)	66.3 (10.8)	2.43 (1.725)
2022	47	27 (57.4%)	62.6 (11.5)	1.62 (1.453)
2023	29	19 (65.5%)	59.8 (13.3)	1.79 (1.84)
Sum	497			
*p*-value		0.422	0.84	0.318

SD, standard deviation; CCI, Charlson comorbidity index. The *p*-values represent the significance of the monotonic trend over 14 years as measured by Spearman’s rank correlation analysis.

**Table 4:** Multivalve procedures during 2010–2023.

Year	All Multivalve	Male (%)	Age, Mean (SD)	CCI, Mean (SD)
2010	6	3 (50.0%)	62.6 (19.3)	3.00 (2.366)
2011	16	4 (25.0%)	68.7 (11.4)	2.75 (1.528)
2012	12	3 (25.0%)	64.8 (13.5)	2.58 (1.782)
2013	21	9 (42.9%)	63.8 (12.7)	2.10 (1.67)
2014	24	15 (62.5%)	66.3 (14.0)	3.17 (1.81)
2015	20	14 (70.0%)	64.2 (15.8)	2.55 (2.139)
2016	21	10 (47.6%)	66.6 (12.0)	2.43 (1.434)
2017	10	5 (50.0%)	68.7 (11.5)	2.40 (1.506)
2018	9	5 (55.6%)	72.6 (9.6)	3.89 (2.088)
2019	14	13 (92.9%)	71.1 (9.0)	2.43 (1.399)
2020	14	8 (57.1%)	64.2 (9.0)	2.36 (1.865)
2021	23	14 (60.9%)	62.9 (12.7)	1.83 (1.466)
2022	13	6 (46.2%)	66.6 (10.9)	2.23 (1.301)
2023	21	12 (57.1%)	66.0 (7.7)	2.24 (1.758)
Sum	224			
*p*-value		0.127	0.553	0.028

SD, standard deviation; CCI, Charlson comorbidity index. The *p*-values represent the significance of the monotonic trend over 14 years as measured by Spearman’s rank correlation analysis.

## Data Availability

Restrictions apply to the datasets. The datasets presented in this article are not readily available because data from this study are ethically and legally restricted by the Institutional Review Board of Tel Aviv Sourasky Medical Center to prevent compromise of patient confidentiality. Requests to access the datasets should be directed to Dr. Shmuel Kivity, Chairman of the Tel Aviv Sourasky Medical Center Institutional Review Board (IRB)/Ethics (Helsinki) Committee at: allergy@tlvmc.gov.il.
